# IL-15 Enhances Activation and IGF-1 Production of Dendritic Epidermal T Cells to Promote Wound Healing in Diabetic Mice

**DOI:** 10.3389/fimmu.2017.01557

**Published:** 2017-11-24

**Authors:** Yangping Wang, Yang Bai, Yashu Li, Guangping Liang, Yufeng Jiang, Zhongyang Liu, Meixi Liu, Jianlei Hao, Xiaorong Zhang, Xiaohong Hu, Jian Chen, Rupeng Wang, Zhinan Yin, Jun Wu, Gaoxing Luo, Weifeng He

**Affiliations:** ^1^State Key Laboratory of Trauma, Burn and Combined Injury, Institute of Burn Research, Southwest Hospital, The Third Military Medical University, Chongqing, China; ^2^Wound Healing Center, Chinese PLA General Hospital, Medical School of Chinese PLA, Beijing, China; ^3^Department of Plastic Surgery, The First Affiliated Hospital of Zhengzhou University, Zhengzhou, Henan, China; ^4^Guangdong Province Key Laboratory of Molecular Immunology and Antibody Engineering, Biomedical Translational Research Institute, Jinan University, Guangzhou, China; ^5^Chongqing Key Laboratory for Disease Proteomics, Chongqing, China; ^6^Department of Dermatology, Xinqiao Hospital, The Third Military Medical University, Chongqing, China

**Keywords:** wound healing, dendritic epidermal T cell, IL-15, IGF-1, diabetes mellitus, type 1

## Abstract

Altered homeostasis and dysfunction of dendritic epidermal T cells (DETCs) contribute to abnormal diabetic wound healing. IL-15 plays important roles in survival and activation of T lymphocytes. Recently, reduction of epidermal IL-15 has been reported as an important mechanism for abnormal DETC homeostasis in streptozotocin -induced diabetic animals. However, the role of IL-15 in impaired diabetic wound healing remains unknown. Here, we found that, through rescuing the insufficient activation of DETCs, IL-15 increased IGF-1 production by DETCs and thereby promoted diabetic skin wound repair. Regulation of IGF-1 in DETCs by IL-15 was partly dependent on the mTOR pathway. In addition, expression of IL-15 and IGF-1 were positively correlated in wounded epidermis. Together, our data indicated that IL-15 enhanced IGF-1 production by DETCs to promoting diabetic wound repair, suggesting IL-15 as a potential therapeutic agent for managing diabetic wound healing.

## Introduction

Dendritic epidermal T cells (DETCs) uniformly express an invariant Vγ5Vδ1 TCR (1) and reside in murine epidermis. DETCs are required for efficient wound repair, in which IGF-1 as one of key factors plays critical roles (2–5). The altered homeostasis and dysfunctions of DETCs contribute to impaired diabetic wound healing (6, 7). Further, a notable reduction of epidermal IGF-1 production, which is majorly secreted by DETCs, has been reported in streptozotocin (STZ)-induced diabetic animals (8). Moreover, addition of rIGF-1 onto wound bed significantly improves defects of diabetic wound healing (9–12). Thus, reduction of IGF-1 by DETCs contributes to defective diabetic wound repair. However, the underlying mechanisms for impaired IGF-1 production by DETCs in diabetes remain unknown.

Activation of DETCs is requisite for IGF-1 production by DETCs. TCR signaling is crucial for activation of γδ T cells. TCR on DETCs senses unidentified ligands on damaged keratinocytes to be activated after skin injury (13). Besides TCR signaling, other co-stimulatory molecules (such as NKG2D, JAML, and Skint1) have been recently shown to contribute to DETCs activation (14–17). Further, IL-2 and IL-15 as T cell growth factors are critical for fully activation of T lymphocytes. IL-15 is required for the development and homeostasis of DETCs in the epidermis (18–20). Recently, reduced epidermal IL-15 production has been shown to participate in altering DETC homeostasis of STZ-induced diabetic animals (8). However, the precise roles of IL-15 in the abnormal activation of DETCs and delayed skin wound closure in diabetic animals have not yet been clarified.

The Akt/mTOR pathway is the central regulator of metabolism that plays a pivotal role in the pathogenesis of diabetes (21–23). The mTOR pathway also plays a critical role in regulating IGF-1 production by DETCs (24). But the role of mTOR pathway in IL-15-mediated regulation of IGF-1 production by DETCs needs to be further investigated.

Keratinocytes are a major source of IL-15 in the epidermis (18). IGF-1 directly stimulates keratinocytes to produce IL-15 partly through an mTOR-dependent pathway (8). Furthermore, the productions of IGF-1 and IL-15 in the epidermis exhibit similar kinetics in STZ-induced diabetic mice and rapamycin-treated non-diabetic animals (25), suggesting that keratinocyte-derived IL-15 and DETC-derived IGF-1 closely correlated with each other in epidermis. However, the exact relationship between keratinocyte-derived IL-15 and DETC-derived IGF-1 in epidermis remains to be clarified.

In the present study, we showed that reduced IL-15 production contributed to impaired activation and pro-healing functions of DETCs, and subsequently delayed skin wound healing in STZ-induced diabetic mice. Further, we revealed that IL-15 regulated IGF-1 expression in DETCs partly by activation of the mTOR pathway, which favored in turn wound healing in diabetic mice.

## Results

### Impaired Activation and IGF-1 Production of DETCs in Diabetic Mice after Skin Injury

**Figure 1:**
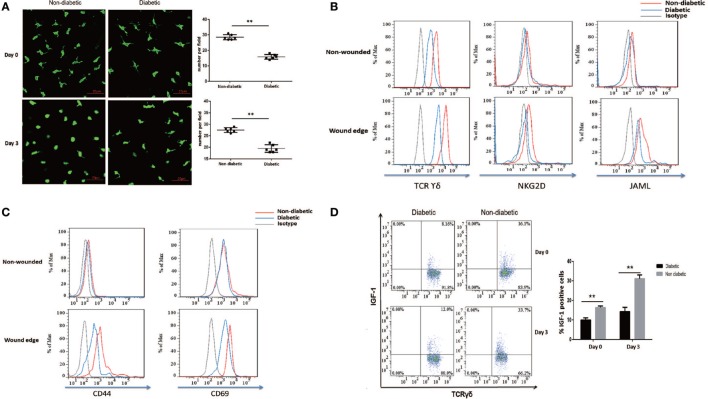
Impaired activation and IGF-1 production of dendritic epidermal T cells (DETCs) in diabetic mice after skin injury. The sex- and age-matched B6 mice were administered streptozotocin (STZ) or vehicle, daily for 6 days. Full-thickness wounds were generated using a sterile 4-mm punch tool at 3 weeks after STZ treatment. Non-wounded and wounded (day 3 after skin injury) epidermis tissues were collected from two to three mice for further analysis. **(A)** The morphology (left panel) and number per field (right panel) of DETCs around the wounds in diabetic mice and non-diabetic controls were analyzed by means of IF (FITC-conjugated anti-γδ TCR staining). **(B–D)** The levels of TCR δ, co-stimulatory molecules (NKG2D, and JAML) **(B)**, T-cell activation markers (CD44 and CD69) **(C)**, and IGF-1 production **(D)** were analyzed in DETCs surrounding the wound edges in STZ-induced diabetic mice and non-diabetic controls by means of FACS. Error bars represent mean ± SD. All data were representative of three individual experiments. *p*-Value was calculated using Student’s unpaired *t*-test (**p < 0.01).

Through secreting IGF-1 and KGF-1, DETCs contribute to efficient wound healing (3, 4, 26, 27). Our previous study reported that diabetic mice exhibited decreased levels of IGF-1 and a substantial reduction of DETCs in both intact and wounded epidermis compared with vehicle-treated controls (9). In line with these results, a significantly decreased number of DETCs in both of intact and wounded epidermis was observed in diabetic mice as compared to that in non-diabetic controls (Figure 1A). DETCs in non-diabetic rather than diabetic animals showed a significantly morphological change (3) (retraction of dendrites and acquisition of a round morphology, Figure 1A) after skin injury, suggesting an impaired activation of diabetic DETCs. Further, we showed that STZ-induced diabetic mice significantly decreased expression levels of TCR and co-stimulatory molecules (NKG2D and JAML) on DETCs in both intact and wounded epidermis, as compared to non-diabetic controls (Figure 1B). Downregulation of TCR, NKG2D, and JAML could have negative implications for T-cell activation (28–30). As compared to non-diabetic controls, diabetic mice showed a comparable expression of T-cell activation markers (CD44 and CD69) on DETCs in intact epidermis, whereas a significantly decreased levels in wounded epidermis (Figure 1C). In addition, IGF-1 production of DETCs in both of intact and wounded epidermis was significantly decreased in diabetic mice compared with non-diabetic controls (Figure 1D). Together, these results indicated that activation of DETCs in the epidermis surrounding the wound edge was impaired in diabetic mice.

### Decreased Epidermal IL-15 Expression Contributes to Impaired DETC Activation in Diabetic Mice

**Figure 2:**
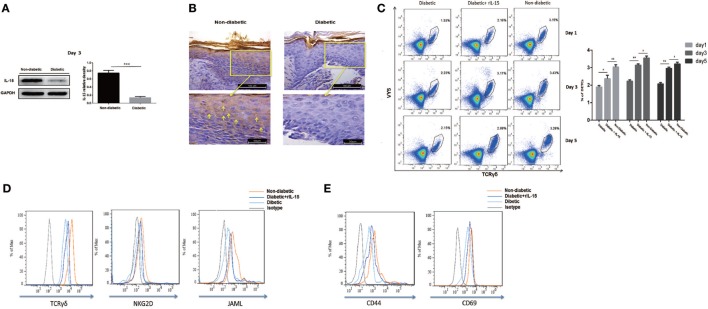
Decreased epidermal IL-15 expression contributes to impaired dendritic epidermal T cell (DETC) activation in diabetic mice. The sex- and age-matched B6 mice were administered streptozotocin (STZ) or vehicle, daily for 6 days. Full-thickness wounds were generated using a sterile 4 mm punch tool at 3 weeks after STZ treatment. **(A,B)** On day 6 post-wounding, the expression of IL-15 in epidermis around wound edge was analyzed by WB **(A)** and IHC **(B)**. **(C–E)** Application of rIL-15 or vehicle control onto wound bed of STZ-induced diabetic mice daily for 6 days upon skin injury. **(C)** On day 1, 3, and 5 post-wounding, the frequency of DETCs in epidermis around wound edge was analyzed by means of FACS. **(D,E)** On day 3 post-wounding, the levels of TCR δ and co-stimulatory molecules (NKG2D and JAML) **(D)**, T-cell activation markers (CD44 and CD69) **(E)** were analyzed by means of FACS. All data represent at least three independent experiments. The values were calculated as the mean ± SD. *p*-Value was calculated by Student’s unpaired *t*-test **(A)** or One-way ANOVA with Bonferroni’s multiple comparison test **(C)** (*p < 0.05, **p < 0.01, ***p < 0.001).

IL-15 is a very important growth factor for efficient activation of T cell in peripheral. DETCs in both intact and wounded epidermis highly express IL-15 receptor alpha (IL-15Rα, CD215), but not IL-2 receptor alpha (CD25), on their surface (Figure S1 in Supplementary Material). It suggested that IL-15 played more important roles in activating DETCs than IL-2 did. STZ-induced diabetic mice exhibited a markedly reduced IL-15 production in epidermis (WB, Figure 2A; IHC, Figure 2B) and IL-15 receptor alpha (IL-15Rα) levels on DETCs (Figure S1 in Supplementary Material) around wound edge as compared to control animals. Further, addition of rIL-15 onto wound bed of diabetics significantly increased the frequency (Figure 2C) and number (Figure S2 in Supplementary Material) of DETCs in epidermis at wound edge, and up-regulated the expression of TCR, co-stimulatory molecules (NKG2D and JAML) (Figure 2D), and T-cell activation markers (CD44 and CD69) (Figure 2E) on DETCs around wound edge at day 3 after skin injury. These findings suggested that, in STZ-induced diabetic mice, reduced epidermal IL-15 production contributed to the abnormal activation DETCs.

### IL-15 Rescues the Defective IGF-1 Expression and Wound Healing of Diabetic Mice

**Figure 3:**
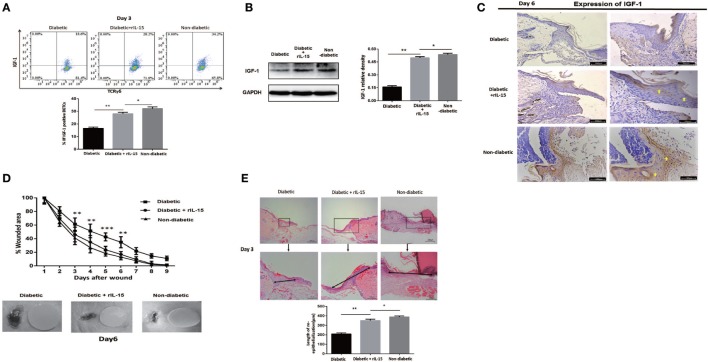
IL-15 rescues the defective IGF-1 expression and wound healing of diabetic mice. The sex- and age-matched B6 mice were administered streptozotocin (STZ) or vehicle, daily for 6 days. Full-thickness wounds were generated using a sterile 4-mm punch tool at 3 weeks after STZ treatment. Application of rIL-15 or vehicle control onto wound bed of STZ-induced diabetic mice daily for 6 days upon skin injury. **(A)** On day 6 post-wounding, IGF-1 production by dendritic epidermal T cells was analyzed by means of FACS. **(B,C)** On day 6 post-wounding, the epidermal IGF-1 production was examined by WB **(B)** and IHC **(C)**. **(D,E)** Wound-closure kinetics was measured over time in wound model with contraction (*n* = 9–11) **(D)**. On day 3 post-wounding, re-epithelialization in wound model without contraction was analyzed by HE (*n* = 5–7) **(E)**. All data represent at least three independent experiments. The values were calculated as the mean ± SD. *p*-Value was calculated by Student’s unpaired *t*-test **(D)** or One-way ANOVA with Bonferroni’s multiple comparison test **(A,B,E)** (*p < 0.05, **p < 0.01, ***p < 0.001).

We further investigated the precise role of IL-15 in impaired diabetic wound healing. The results showed that application of rIL-15 significantly enhanced IGF-1 production in diabetic DETCs (Figure 3A) and epidermis (Figures 3B,C) around wound edge, and subsequently promoted diabetic skin wound closure (wound model with contraction, Figure 3D) and re-epithelialization [wound model without contraction (31), Figure 3E]. In addition, blocking IL-15 with rIL-15Rα notably decreased epidermal IGF-1 secretion in non-diabetic animals (Figures 5E,F). Interestingly, the expression levels of IL-15Rα rather than CD25 on diabetic DETCs could be significantly elevated by addition of rIL-15 (Figure S3 in Supplementary Material). Thus, IL-15 could rescue the defective IGF-1 expression and wound healing of STZ-induced diabetic mice.

### IL-15 Enhances TCR-Induced IGF-1 Production by DETCs Partly through an mTOR-Dependent Pathway

**Figure 4:**
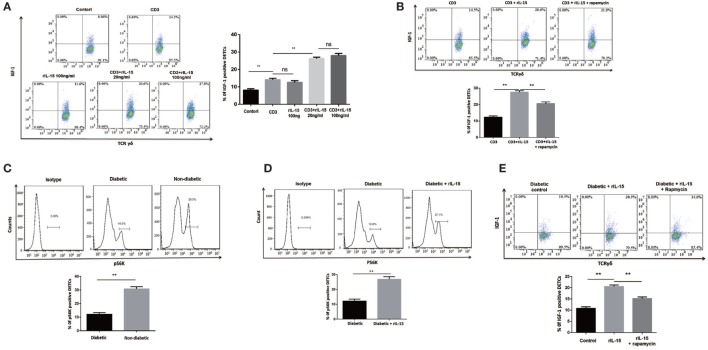
IL-15 enhances TCR-induced IGF-1 production by dendritic epidermal T cells (DETCs) partly through an mTOR-dependent pathway. **(A,B)** DETCs were isolated from epidermal cells around wound of B6 mice at day 3 after skin injury by means of MACS (purity >95%). **(A)** The DETCs were stimulated with anti-CD3 and rIL-15 alone or together for 24 h, and IGF-1 production by DETCs were detected by FACS. **(B)** The DETCs were stimulated with anti-CD3 plus rIL-15 in the presence of rapamycin or control vehicle for 24 h, then IGF-1 production by DETCs were detected by FACS. **(C)** The expression of phosphorylated S6 kinase (pS6K) in DETCs at wound edge of streptozotocin (STZ)-induced diabetic animals or non-diabetic controls was examined on day 3 after skin injury by FACS. **(D,E)** Application of rIL-15 or control vehicle onto wound bed of STZ-induced diabetic mice daily for 3 days and then the expression of pS6K **(D)** and IGF-1 production **(E)** in DETCs at wound edge was examined on day 3 after skin injury by FACS. All data represent at least three independent experiments. The values were calculated as the mean ± SD. *p*-Value was calculated by Student’s unpaired *t*-test **(C,D)** or One-way ANOVA with Bonferroni’s multiple comparison test **(A,B,E)** (*p < 0.05, **p < 0.01).

IL-15 has been well demonstrated to be required for DETCs homeostasis. However, the precise role of IL-15 in IGF-1 production of DETCs has not been fully clarified. Here, we showed that high dose rIL-15 (100 ng/ml) slightly increased IGF-1 production in DETCs freshly isolated from wound area by MACS. But low dose rIL-15 (20 ng/ml) significantly enhanced IGF-1 production in the DETCs upon anti-CD3 stimulation (Figure 4A). It indicated that TCR signaling is required for IL-15 mediated enhancement of IGF-1 by DETCs.

Impaired mTOR signaling has been exhibited in diabetic epidermis. Others and our previous studies showed that mTOR signaling was deeply involved in regulating IGF-1 production in epidermis and DETCs (8). Now, we showed that rapamycin (an inhibitor of mTOR signaling) notably inhibited IGF-1 production in DETCs, which were freshly isolated from wound area by MACS, in the presence of rIL-15 plus anti-CD3 stimulation *in vitro* (Figure 4B), suggesting that mTOR was involved in IL-15-mediated upregulation of IGF-1 production from DETCs. Further, the expression of phosphorylated S6 kinase, an mTOR downstream kinase, in DETCs around wound edge was significantly decreased in STZ-induced diabetic mice compared to non-diabetic controls (Figure 4C). Moreover, addition of rIL-15 onto wound bed of diabetic animals markedly augmented activation of mTOR signaling in DETCs (Figure 4D). Application of rapamycin onto wound bed of diabetic animals markedly attenuated rIL-15-mediated enhancement of IGF-1 production by DETCs (Figure 4E). Collectively, in STZ-induced diabetic animals, impaired mTOR signaling contributed to reduction of IGF-1 by DETCs, and IL-15 enhanced activation of mTOR signaling to increase IGF-1 production by DETCs.

### IL-15 and IGF-1 Regulate Each Other in the Wounded Epidermis and in Cocultures of Keratinocytes with Activated DETCs

**Figure 5:**
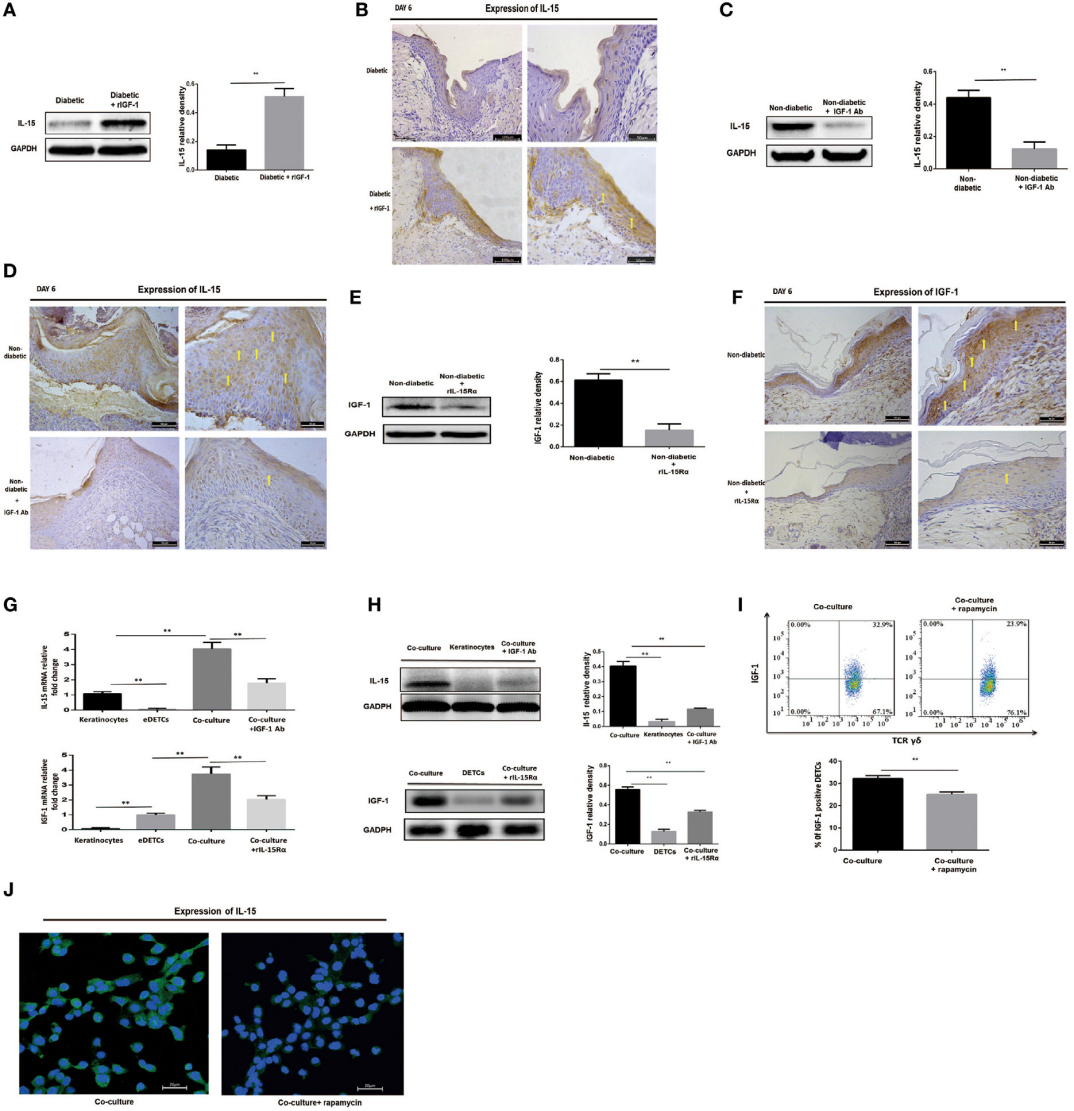
IL-15 and IGF-1 regulate each other in the wounded epidermis and in cocultures of keratinocytes with activated dendritic epidermal T cells (DETCs). **(A,B)** Application of rIGF-1 or vehicle control onto wound bed of streptozotocin (STZ)-induced diabetic mice daily for 6 days, then epidermal IL-15 production was examined by WB **(A)** and IHC **(B)**. **(C,D)** Application of IGF-1 neutralizing antibody or isotype IgG control onto wound bed of B6 mice daily for 6 days, then epidermal IL-15 production was examined by WB **(C)** and IHC **(D)**. **(E,F)** Application of rIL-15Rα or isotype IgG control onto wound bed of B6 mice daily for 6 days, and then epidermal IGF-1 production was examined by WB **(E)** and IHC **(F)**. **(G–J)** DETCs were isolated from B6 mice and expanded with Con A stimulation for 4–6 weeks. The expanded DETCs (eDETCs) (purity >95%) were rested without Con A for 2 weeks before further study. Primary keratinocytes were isolated from newborn male B6 mice and CD11c^+^ cells were depleted by means of MACS (purity >95%). **(G,H)** The eDETCs cocultured with primary keratinocytes (1:2) in the presence of IGF-1 neutralizing antibody or rIL-15α for 24 h, and IL-15 and IGF-1 expression were detected by real-time PCR **(G)** and Western blot **(H)**. **(I,J)** The eDETCs cocultured with primary keratinocytes (1:2) in the presence of rapamycin for 24 h, and IL-15 and IGF-1 expression were detected by FACS **(I)** and IF **(J)**. All data represent at least three independent experiments. The values were calculated as the mean ± SD. *p*-Value was calculated by Student’s unpaired *t*-test **(A,C,E,I)** or One-way ANOVA with Bonferroni’s multiple comparison test **(G,H)** (*p < 0.05, **p < 0.01).

As mentioned above, IL-15 enhances DETC activation to produce IGF-1 upon TCR stimulation. IGF-1 directly stimulates keratinocytes to produce IL-15, suggesting that DETC-derived IGF-1 and keratinocyte-derived IL-15 might promote each other’s production. Indeed, administration of rIGF-1 or rIL-15 onto wound bed also enhanced the secretion of IL-15 (Figures 5A,B) or IGF-1 (Figures 3B,C) in the epidermis around the wounds in diabetic animals. Application of IGF-1 neutralizing antibody or rIL-15Rα onto the wound bed markedly weakened epidermal IL-15 (Figures 5C,D) or IGF-1 (Figures 5E,F) production around wound in non-diabetic mice. To further investigate the precise relationship between keratinocyte-derived IL-15 and DETC-derived IGF-1, we cocultured the per-expanded DETCs (eDETCs), which are ready to secrete IGF-1 without TCR engagement, and primary keratinocytes isolated from newborn B6 mice. Compared to eDETCs or keratinocytes cultured alone, cocultured eDETCs and keratinocytes significantly increased levels of secreted IL-15 and IGF-1 at both mRNA (Figure 5G) and protein (Figure 5H) levels. Blockade of IGF-1 or IL-15 by neutralizing antibodies or rIL-15Rα markedly attenuated the increase of IL-15 and IGF-1 productions (Figures 5G,H), respectively, in the coculture system. However, cocultured freshly isolated DETCs with primary keratinocytes failed to significantly increase IGF-1 and IL-15 productions, as compared to the DETCs or the keratinocytes cultured alone, respectively (data not shown). Thus, a positive correlation, but not a loop, existed between keratinocyte-derived IL-15 and DETC-derived IGF-1.

Impaired mTOR pathway in diabetic or rapamycin-treated mice contributed to reduction of IGF-1 and IL-15 in epidermis, suggesting that the mTOR pathway regulates the interaction between keratinocytes and DETCs. To address this issue, we cocultured eDETCs with primary keratinocytes in the presence of rapamycin. In line with these results of epidermal cells, the productions of IGF-1 in DETCs (Figure 5I) and IL-15 in keratinocytes (Figure 5J) at single-cell level were downregulated by rapamycin. Moreover, increased release of IGF-1 or IL-15 in the epidermis around the wounds, which was mediated by addition of rIL-15 or rIGF-1 onto the wound bed, respectively, was markedly attenuated in rapamycin-treated B6 mice (Data not shown). Furthermore, application of rIGF-1 onto wound bed significantly increased the activation of the mTOR pathway in DETCs at wound margin of diabetic animals (data not shown). Collectively, DETC-derived IGF-1 and keratinocyte-derived IL-15 were mutually enhanced in wounded epidermis, in which mTOR signaling was partly involved.

## Discussion

Activation of DETCs around wound edge is required for their proliferation and IGF-1 secretion, which plays an important role in wound healing (2–4). Altered homeostasis and dysfunctions of DETCs are one of important reasons for abnormal diabetic wound healing (9). IL-15 has been reported to play crucial roles in development and homeostasis of DETCs (19, 32). Recently, we showed that reduction of epidermal IL-15 contributed to the altered homeostasis of DETCs in STZ-induced diabetic mice (8). In this study, we further revealed that impaired epidermal IL-15 production was a critical reason for insufficient activation and reduced IGF-1 production of DETCs in diabetic animals. Addition of exogenous IL-15 onto wound bed could rescue the defective activation and IGF-1 expression of diabetic DETCs, and thereby promote diabetic wound healing. Thus, we for the first time revealed an unrecognized role of IL-15 in promoting diabetic wound healing.

Differing from αβ T cells, γδ T cells could be directly activated by TCR signaling without co-stimulatory signaling help (33). Besides TCR signaling, IL-2, and/or IL-15 as T cell growth factors are also required for survival and fully activation of γδ T cells. Interestingly, epidermal IL-15, but not IL-2, has been well demonstrated to play critical roles in DETC homeostasis (14, 34). Moreover, we here showed that IL-15Rα (CD215), but not IL-2Rα (CD25), was highly expressed on surface of DETCs in both of wounded and non-wounded epidermis. Together with facts that the activated DETCs are failures to produce IL-2 (Data not shown), leading us to believe that IL-15 is more important than IL-2 for homeostasis and efficient activation of DETC at early stage of wounding. In line with the notion, application of exogenous IL-15 improved the insufficient activation and IGF-1 production of DETCs in STZ-induced diabetic mice. Therefore, impaired epidermal IL-15 production was an important reason to result in dysfunctional DETCs in diabetes.

Epidermal IL-15 is predominantly derived from keratinocytes and Langerhans cells (35, 36). Our previous studies showed that keratinocyte-derived IL-15 could be regulated by IGF-1, and a similar kinetics of epidermal IGF-1 and IL-15 production has been displayed in STZ- or rapamycin-treated mice (8). Here, we further confirmed a positive correlation between epidermal IL-15 and IGF-1 production at wound edge after skin injury. Addition of rIL-15 or rIGF-1 onto wound bed increased, whereas blocking of IL-15 or IGF-1 decreased, epidermal IGF-1 or IL-15 production, respectively. Although these findings suggested that DETC-derived IGF-1 and keratinocyte-derived IL-15 may positively feedback with one another, freshly isolated DETCs cocultured with primary keratinocytes failed to significantly increase the production of IGF-1 and IL-15 (data not shown). A rational explanation is inability of IL-15 to directly stimulated IGF-1 production by DETCs in the absence of TCR engagement. Indeed, addition of exogenous IL-15 failed to significantly increase IGF-1 production by DETCs *in vitro*. However, eDETCs, which were expanded with Con A *in vitro* and ready to produce IGF-1 without TCR engagement, cocultured with keratinocytes significantly enhanced IGF-1 production by DETCs and IL-15 production from keratinocytes, and the enhancements were significantly attenuated by blocking of IL-15 or IGF-1 respectively. Collectively, although a positive correlation was form between IL-15 and IGF-1 in wounded epidermis (where TCR ligands are made available to DETCs), keratinocyte-derived IL-15 failed to directly stimulate DETCs to produce IGF-1 for forming an IL-15-IGF loop when TCR signaling is lacking.

The mTOR signaling is the central regulator of metabolism and plays critical roles in survival, proliferation, and functions of DETCs (24). Recently, mTOR pathway has been shown to control IL-15-mediated IGF-1 production by keratinocytes *in vitro*, and regulate both of epidermal IL-15 and IGF-1 secretion *in vivo*. Here, we further identified that the mTOR pathway was partially involved in regulating IL-15-mediated IGF-1 production by DETCs. Application of rapamycin markedly attenuated IL-15-mediated enhancement of IGF-1 by DETCs *in vitro* and *in vivo* in response to diabetic wounding. It is noteworthy that IL-15 and IGF-1 could activate mTOR signaling pathway in DETC and keratinocyte, respectively, and the abnormal activation of the mTOR pathway in diabetic DETCs around wound edge could be enhanced by addition of rIL-15 or rIGF-1 *in vivo*. This implies that, in diabetes setting, impaired mTOR pathway and weakened productions of IL-15 and IGF-1, exhibit mutual effects and may reach a low-level equilibrium state, an important mechanism that causes dysfunction of DETC and defective wound repair.

IL-15 enhanced DETC pro-healing function in the presence of TCR engagement. This provides a rational explanation why blocking of IL-15 has no significant affection on activation of DETCs and wound healing in non-diabetic mice (data not shown), while TCR signaling in DETC is severely impaired in diabetes setting and IL-15 will become critical for DETC activation under this condition. Furthermore, the expressions of other important co-stimulatory molecules, such as JAML and NKG2D, on DETCs also notably decreased in diabetic animals. Intriguingly, addition of rIL-15 onto wound bed could remarkably enhance expressions of the co-stimulatory molecules on DETCs in STZ-induced diabetic mice. In addition, exogenous IL-15 also significantly increased IL-15Rα expression on diabetic DETCs *in vivo*. These findings suggested that, in a diabetes setting, IL-15 might elevate the expression of TCR, co-stimulatory molecules, and IL-15Rα on DETCs to further enhance activation and pro-healing function of DETCs in diabetic animals.

It is worth noting that IGF-1 receptor (IGF1R) played a crucial role in keratinocyte homeostasis and migration for efficient skin wound healing. Impaired IGF1R expression on epidermal cells in diabetic animals resulted in defective wound repair. Addition of exogenous IL-15 could elevate IGF1R levels on epidermal cells around wound edge in STZ-induced diabetic mice (Figure S4 in Supplementary Material), which might further promote diabetic wound healing. However, the underlying mechanism need to be further investigated.

T lymphocytes in murine epidermis are exclusively consisted of DETCs. Though reduction of DETCs in diabetic mice, few αβ T cells were observed to infiltrate into epidermis after wounding (Figure S5 in Supplementary Material). It suggested that, at early stage of wounding, αβ T cells did not participate in regulating re-epithelialization of skin wound healing. However, differing from murine, αβ T cells were an important component of human epidermal immune system. The roles of these cells in human skin wound healing need to be clarified in near future.

In addition, impaired inflammation has been demonstrated to contribute to defective diabetic wound healing (37). Vγ4 T cells as major source of IL-17A played critical role in skin inflammation at early stage of skin injury (38). Application of Vγ4 T cells onto wound bed promoted wound healing of STZ-induced diabetic mice (39). It is reasonable to assume that IL-15, an important T-cell growth factor, enhances IL-17A production by Vγ4 T cells to promoting diabetic wound healing. The issue needs to be confirmed in near future.

In summary, our results suggest that reduction of epidermal IL-15 is one of the factors that leads to dysfunction of DETC in diabetic animals after skin injury. Administration of exogenous IL-15 could improve insufficient activation, altered homeostasis, and dysfunction of DETCs to promote diabetic wound healing. Thus, our study provided evidence that exogenous IL-15 could be considered as new approach to promote diabetic wound healing.

## Materials and Methods

### Mice

C57BL/6 (B6) mice were purchased from the Experimental Animal Center in the Third Military Medical University in Chongqing, China. All mice were housed under specific pathogen-free conditions at the Southwest Hospital Experimental Center (Chongqing, China) and given food and water *ad libitum*. Male B6 mice aged 6–8 weeks were used for establishing STZ-induced diabetic animal model. STZ-induced diabetic male mice and non-diabetic controls aged 9–11 weeks were used for further wounding assays. All experiments were performed under conventional animal raising conditions in conformity with ethical guidelines and approved by the Animal Ethics Committee of the Third Military Medical University.

### STZ-Induced Diabetic Animal Model

B6 male mice with 6–8 weeks of age were injected i.p. with 150 µl streptozotocin (STZ) (100 mg/kg, Sigma-Aldrich, USA) or vehicle control for 6 consecutive days. Blood glucose levels were measured in the venous blood from non-fasted animals using a glucometer (Ange, CHN). Mice were evaluated everyday and were considered diabetic when sustained blood glucose levels exceeded 250 mg/dl. These diabetic mice were used for further wounding model at 3 weeks after the initiation of STZ treatment.

### Wounding Procedure

Wounding was performed on age- and sex-matched B6 controls and STZ-induced diabetic mice (9–11 weeks, male), which were anesthetized with 1% sodium pentobarbital (5–10 µl/g of body weight, Sigma-Aldrich, GER). Briefly, the dorsal surface of the mouse was shaved and the back skin and panniculus carnosus were pulled up. Thereafter, 2 or 4 sets of sterile full-thickness wounds were established using a sterile 4-mm punch tool. Wound area was recorded daily by means of macroscopic digital photographs. Based on the picture, the area of the wound was measured using IPP 6.0 software.

For wound model without contraction, the biological membrane (NPWT-1, Negative pressure wound therapy kit, China) was glued with the adhesive dressings immediately onto the surface of the wound before contraction, and mice caged individually. At day 3 after skin injury, the wound tissue, with adjacent normal skin, was carefully biopsied and stained with HE for histological analysis. The lengths of the epithelial tongue (newly generated epidermis) were measured with IPP 6.0 software. The length of the epithelial tongue was defined as the distance between the advancing edges of the epidermal keratinocytes and the hair follicles in non-wounded skin (31).

In some experiments, 100 ng of recombinant IGF-1 (R&D Systems, USA), 100 ng recombinant IL-15 (R&D Systems, USA), 10 µg mouse IGF-1 neutralization antibody (R&D Systems, USA), 5 µg recombinant mouse IL-15 R alpha Fc Chimera protein (rIL-15Rα, R&D system, USA), or control buffer alone was injected into each of wound sites post-wounding subcutaneously and daily thereafter for 6 days.

For rapamycin administration, 6- to 8-week male B6 mice were injected intraperitoneally with 200 µl rapamycin (4 mg/kg/day, Selleck Chemicals, USA), which dissolved in 0.2% carboxymethyl cellulose and 0.25% Tween 80 (Sigma-Aldrich, GER), or with vehicle control daily for 6 days after wounding.

### Isolation of Epidermal Cells

The whole skin at wound edge, which was less than 5 mm away from the border of wound, was collected. Each group of skin tissue at wound edge was collected from at least three mice for analysis of real-time PCR, western blotting (WT), and FACS. The subcutaneous tissue was wiped off from the wound edge and was then cut into 0.5 cm × 1 cm pieces. After incubating with 5 mg/ml dispase II for 1–2 h at 37°C, epidermal sheets were separated from wound edge. Epidermal single cells were dissociated with a 0.3% Trypsin/GNK solution. Enzymatic dissociation was terminated by means of adding twofold of 10% FBS 1640 medium. Epidermal single-cell suspensions were filtered through a 70-µm strainer. For surface staining, epidermal single-cell suspensions were washed and suspended to a concentration of 1 × 10^6^ cells/ml with PBS.

### Coculture of eDETCs and Keratinocytes

Expanded DETCs were prepared as described previously [Uchida et al. (40)] with some modification. Epidermal sheets were separated from the dorsal skin of 6- to 8-week B6 male mice by incubation with 5 mg/ml dispase II (Roche, GER) for 2 h at 37°C. After enzymatic dissociation with 0.3% Trypsin/GNK solution, epidermal cells were washed and filtered through a 70-µm strainer and were enriched with Lympholyte-M (Cedarlane Laboratories, CAN). DETC short-term cell lines were generated by culturing the epidermal cells in 48-well plates (1–2 × 10^6^/ml) using RPMI with 10% FBS, 100 M non-essential amino acids (Gibco, USA), 1 mM Na pyruvate (Sigma-Aldrich, GER), 2 mM glutamine, 25 mM HEPES, 50 µM 2-ME (Biosharp, CHN), 10 ng/ml mouse rIL-2 (R&D Systems, Minneapolis, MN, USA), 1 µg/ml Con A (Sigma-Aldrich, GER), 100 U penicillin, and 100 µg streptomycin for 4–6 weeks. The purity of eDETC population was >95% by means of FACS analysis. Further experiments were performed with eDETCs rested for 2 weeks without Con A (Sigma-Aldrich, GER). During the resting period, IL-2 was added for maintaining eDETCs survival.

Primary keratinocytes were isolated from newborn B6 male mice. Epidermal single-cell suspensions were obtained according to the protocol mentioned above. CD11c^+^ cells were depleted from primary keratinocytes according to the manufacturer’s instructions (Miltenyi, GER). The purity of isolated keratinocyte population was >95% by means of FACS analysis.

Primary keratinocytes (8 × 10^5^) cocultured with eDETCs (4 × 10^5^) in RPMI 1640 (GIBCO BRL, USA) supplemented with 10% fetal bovine serum (Hyclone, USA). In some cases, 20 ng/ml rapamycin (Selleck Chemicals, USA), 200 ng/ml mouse IGF-1 neutralization antibody (R&D Systems, USA), or 100 ng/ml recombinant mouse IL-15 R alpha Fc Chimera protein (R&D Systems, USA) were added in the coculture system.

### Flow Cytometry Analysis and Intracellular Cytokine Staining

The following fluorochrome-labeled murine monoclonal antibodies (mAbs) were purchased from BD, Biolegend, eBioscience, Sungene Biotech, and Cell Signaling Technology: CD16/32 (2.3G2), γδ TCR (GL3), Vγ5 (536), CD25 (3C7), CD44 (IM7), CD69 (H1.2F3), NKG2D (CX5), JAML (4E10), CD215 (DNT15Ra), and IGF1R (8864). IGF-1 (H70, Santa Cruz Biotechnology, USA) was stained followed by fluorescent-labeled secondary reagents FITC goat-anti-rabbit mAb (Boster, CN), PE donkey-anti-rabbit mAb (Biolegend, USA). For surface staining, cells were first blocked with anti-CD16/32 mAb for 15 min and then incubated with different cellular surface mAbs for 30 min at 4°C. For further intracellular staining, cells were fixed and permeabilized according to the manufacturer’s recommendations (BD Biosciences, USA). For *ex vivo* detection of intracellular cytokines, epidermal cells were stimulated with 50 ng/ml PMA, 750 ng/ml ionomycin (BD Biosciences, USA), and 100 ng/ml GolgiPlug (BD Biosciences, USA) at 37°C, 5% CO_2_ for 6 h. For *in vitro* stimulation, freshly isolated DETCs or epidermal cells cultured with various doses rIL-15 (R&D system, USA) in the presence of TCR engagement for 24 h and GolgiPlug was added for the last 6 h. Cells were stained as above. Stained cells were detected on Attune Acoustic Focusing Cytometer (Life Technologies, USA) and analyzed with FlowJo software (Tree Star Incorporation, USA).

### Western Blot Analysis

Proteins were extracted from skin cells or tissues as described previously (9). Primary Abs were incubated overnight at 4°C: GAPDH (1:2000, SunGene Biotech, China), IGF-1, IL-15 (1:200, Santa Cruz Biotechnology, USA). Subsequently, the membranes were incubated with HRP-labeled goat anti-mouse Ab or goat anti-rabbit Ab (1:5000, SunGene Biotech, CHN) for 1 h. Blots were analyzed using the western blot ChemiDoc™ XRS detection system (Bio-Rad, USA).

### Immunofluorescence

Epidermal sheets around wound were obtained according to the protocol mentioned above. Epidermal sheets were fixed with 4% paraformaldehyde on ice and then the slides were blocked in 5% goat serum plus 0.1% Triton-X-100 at room temperature, followed by overnight incubation with FITC-conjugated anti-γδ TCR (1:50, Tianjin Sungene Biotech Co. Ltd., CHN) or FITC-conjugated anti-IL-15 (Santa Cruz Biotechnology, USA) at 4°C. Then, the images were captured and photographed using the Leica fluorescent microscope (CTR6000, Leica, GER). The numbers of positive cells were assessed by counting 30 random view fields of per specimen. The results were expressed as the mean number of positive cells ± SE per view field obtained from three to five specimens.

### Immunohistochemistry

Skin sections were deparaffinized and hydrated in xylene and graded alcohol series. Antigen retrieval was conducted by incubating sections in 10 mM citric acid (pH 6) at 95°C for 15 min. Sections were blocked and stained with goat-anti-rabbit serum or 3% BSA with 0.3% Triton X-100 (Sigma-Aldrich, GER) at room temperature for 60 min and followed by primary antibodies: anti-IGF-1 and anti-IL-15 (1:200–400, Abcam, UK) overnight at 4°C. Samples were washed and incubated with secondary antibodies at room temperature for 60 min. Slides were incubated with drops of diaminobenzidine solution (Boster, CN) and counterstained with hematoxylin (Beyotime, CN). Stained sections were examined under Olympus BX51 microscope (Tokyo, Japan).

### Quantitative Real-time RT-PCR

Cultured cells or harvested tissue samples were washed with PBS, and total RNA was extracted with an RNeasy^®^ Mini Kit (QIAGEN, GER) according to the manufacturer’s instructions. The RNA concentration and quality were measured by a DU800 UV/Vis spectrophotometer (Beckman Coulter, USA). The mRNA was reverse transcribed with the First Strand cDNA Synthesis Kit (TOYOBO, JPN). Real-time PCR was performed using SYBR Green PCR Master Mix (TOYOBO, JPN) under the following conditions: 95°C for 2 min followed by 50 repetitive cycles of 95°C for 15 s, 60°C for 15 s, and 72°C for 32 s. The primers used in this study were as follows:

**Table:** 

	Forward primer	Reverse primer
IL-15	5′-GGATTTACCGTGGCTTTGAGTAATGAG-3′	5′-GAATCAATTGCAATCAAGAAGTG-3′
IGF-1	5′-GGACCAAGGGGCTTTTACTT-3′	5′-GCAACACTCATCCACAATGC -3′
GAPDH	5′-CGTGCCGCCTGGAGAAAC-3′	5′-AGTGGGAGTTGCTGTTGAAGTC-3′

Quantification of mRNA levels was conducted in the ABI PRISM 7500 Sequence Detection System (Applied Biosystems, Foster City, CA) following the manufacturer’s protocols. Data were analyzed by the 2^−ΔΔ^ *C*_t_ method, and GAPDH served as an internal control.

### Statistical Analysis

All data were shown as mean ± SD. Statistical differences were calculated by two-tailed unpaired Student’s *t*-test or one-way ANOVE with Bonferroni comparison test on SPSS19.0 software (IBM, USA) and graphs were manufactured by GraphPad Prism software (GraphPad Software, Inc., USA). *p* < 0.05 was considered significant.

## Ethics Statement

All experimental methods described above were conducted in accordance with guidelines for animal care and were approved by the First Affiliated Hospital (Southwest Hospital) of the Third Military Medical University. All experiments were approved by the Laboratory Animal Welfare and Ethics Committee of the Third Military Medical University.

## Author Contributions

WH designed the research and analyzed the data. JW, GLuo, and WH wrote the manuscript. YB, YW, ZL, YL, ML, GLiang, JH, XZ, and XH performed the research. YB, ZL, GLuo, RW, ZY, and WH interpreted the data. YB, YJ, JC, and WH prepared the manuscript.

## Conflict of Interest Statement

The authors declare that the research was conducted in the absence of any commercial or financial relationships that could be construed as a potential conflict of interest.

## Supplementary Material

Figure S1 The levels of CD25 and CD215 on DETCs of intact or wounded epidermis in streptozotocin (STZ)-induced diabetic mice and non-diabetic controls were analyzed by means of FACS.

Figure S2 Application of rIL-15 or vehicle control onto wound bed of streptozotocin (STZ)-induced diabetic mice daily for 6 days upon skin injury. On day 1, 3, and 5 post-wounding, the morphology (left panel) and number per field (right panel) of DETCs around wound edge were analyzed by means of IF.

Figure S3 Application of rIL-15 or vehicle control onto wound bed of streptozotocin (STZ)-induced diabetic mice daily for 6 days upon skin injury. On day 3 post-wounding, the levels of CD25 and CD215 on DETCs around wound edge were analyzed by means of FACS.

Figure S4 The expressions of IGF-1 receptor (IGF1R) on epidermal cells from intact or wounded epidermis of diabetic and non-diabetic mice were showed in right panel. The expression of IGF1R on epidermal cells of diabetic mice, diabetic mice with rIL-15 treatment, and non-diabetic controls at day 3 after skin injury was showed in left panel.

Figure S5 Epidermis-infiltrating αβ T cells in intact or wounded skin of diabetic and non-diabetic mice were detected by FACS.
