# Supplementary figures and images for: IL-15 Enhances Activation and IGF-1 Production of Dendritic Epidermal T Cells to Promote Wound Healing in Diabetic Mice
Source: Front Immunol. 2017 Nov 24;8:1557. doi: 10.3389/fimmu.2017.01557 (PMC5705622; doi:10.3389/fimmu.2017.01557)

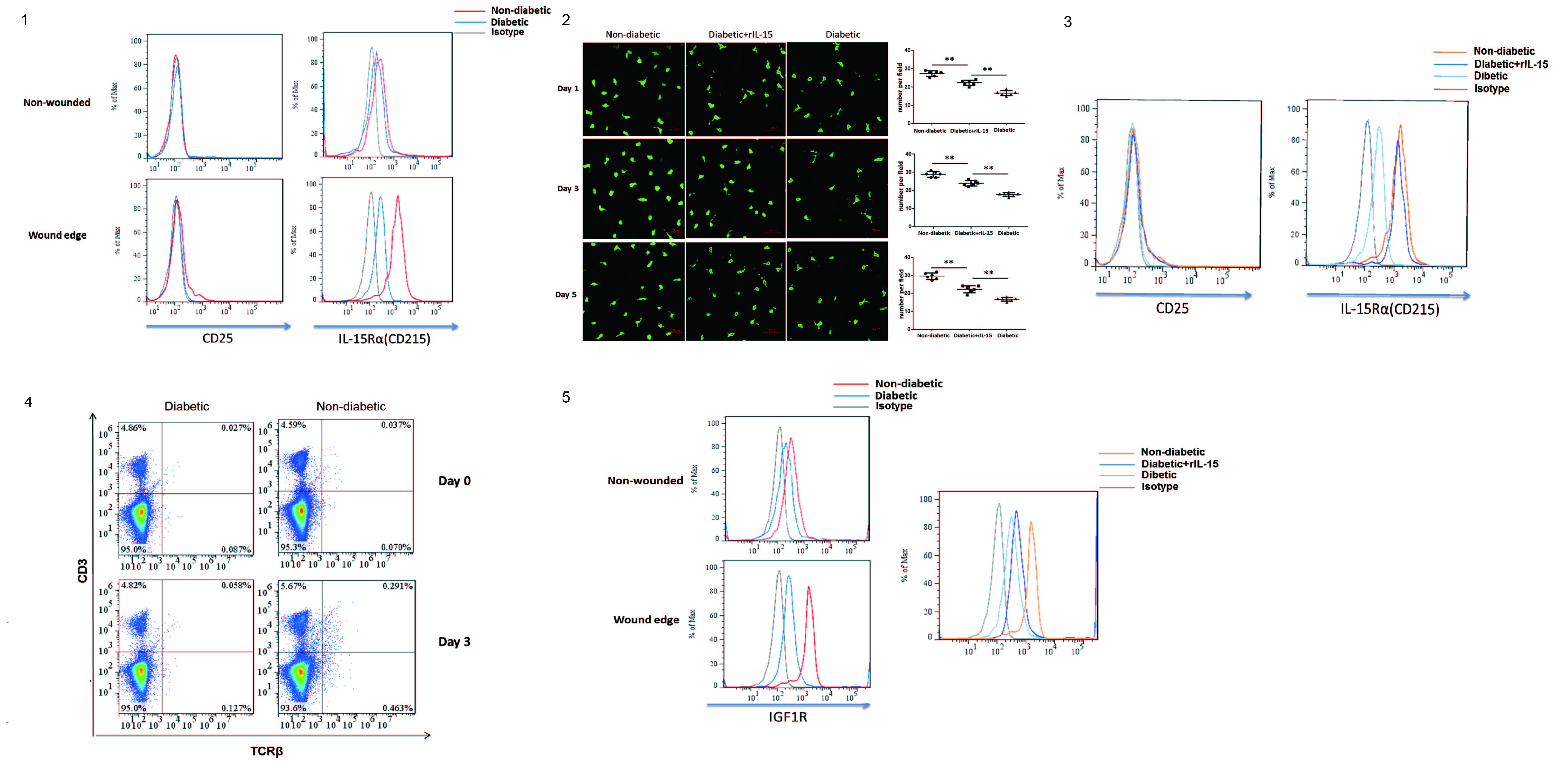

Supplement: Figure S1 — The levels of CD25 and CD215 on DETCs of intact or wounded epidermis in streptozotocin (STZ)-induced diabetic mice and non-diabetic controls were analyzed by means of FACS. [file image_1.jpeg]
